# O14 - Der p 10 as a minor allergen in children sensitised to house dust mites

**DOI:** 10.1186/2045-7022-4-S1-O14

**Published:** 2014-03-14

**Authors:** Isabel Lafuente, Raquel Pina, Sonia Uixera, Rafael Calderon, Isidoro Cortell, Juan Lopez, Antonio Nieto, Angel Mazon

**Affiliations:** 1 Unit of Pediatric Allergy and Pneumology Hospital La Fe, Valencia, Spain

## Objective

To assess the proportion of children with specific IgE to Der p 10 in children with sensitization to house dust mites (HDM).

## Material and methods

We determined specific IgE (ImmunoCAP^®^, Thermo Fisher Scientific) to Der p 1, Der p 2 and Der p 10 simultaneously in children with symptoms of asthma and/or rhinoconjunctivitis who had positive skin tests to HDM. Values <0.35 kUA/L were considered negative.

## Results

We studied 232 children (147 M/85 F) aged 1.25 - 16.7 years. Negative results were found in 50 (21.6%) with Der p 1, 53 (22.8%) with Der p 2, 208 (89.7%) with Der p 10, and 29 with all three (12.5%). There were 24 patients with positive IgE to Der p 10: three of these had IgE values of 6.1 to 11 kUA/L to Der p 10 and negative results to Der p 1 and 2 (0.1 to 0.18), three had much higher IgE to Der p 10 (79.9 to >100) than to Der p 1 or 2 (0,58 to 24.2), and one slightly higher IgE to Der p 10 (2.8) than to Der p 1 or 2 (0.8 and 1.19). The rest of the patients had clearly lower IgE to Der p 10.

## Conclusion

About 10% of patients sensitized to HDM had specific IgE to Der p 10. Sensitization to Der p 10 without sensitization to major allergens Der p 1 and Der p 2 appeared in 1.3% of patients, and serologically more relevant sensitization to Der p 10 in an additional 1.3% of patients. This should be considered when prescribing immunotherapy, as Der p 10 is not standardized in current vaccines.

